# Gelsolin Attenuates Neonatal Hyperoxia-Induced Inflammatory Responses to Rhinovirus Infection and Preserves Alveolarization

**DOI:** 10.3389/fimmu.2022.792716

**Published:** 2022-01-31

**Authors:** Tracy X. Cui, Alexander E. Brady, Ying-Jian Zhang, Christina T. Fulton, Antonia P. Popova

**Affiliations:** Department of Pediatrics, University of Michigan Medical School, Ann Arbor, MI, United States

**Keywords:** gelsolin, prematurity, BPD, rhinovirus, dendritic cells, f-actin, lung inflammation

## Abstract

Prematurity and bronchopulmonary dysplasia (BPD) increase the risk of asthma later in life. Supplemental oxygen therapy is a risk factor for chronic respiratory symptoms in infants with BPD. Hyperoxia induces cell injury and release of damage-associated molecular patterns (DAMPs). Cytoskeletal filamentous actin (F-actin) is a DAMP which binds Clec9a, a C-type lectin selectively expressed on CD103+ dendritic cells (DCs). Co-stimulation of Clec9a and TLR3 induces maximal proinflammatory responses. We have shown that neonatal hyperoxia (a model of BPD) increases lung IL-12+Clec9a+CD103+ DCs, pro-inflammatory responses and airway hyperreactivity following rhinovirus (RV) infection. CD103+ DCs and Clec9a are required for these responses. Hyperoxia increases F-actin levels in bronchoalveolar lavage fluid (BALF). We hypothesized that the F-actin severing protein gelsolin attenuates neonatal hyperoxia-induced Clec9a+CD103+ DC-dependent pro-inflammatory responses to RV and preserves alveolarization. We exposed neonatal mice to hyperoxia and treated them with gelsolin intranasally. Subsequently we inoculated the mice with RV intranasally. Alternatively, we inoculated normoxic neonatal mice with BALF from hyperoxia-exposed mice (hyperoxic BALF), RV and gelsolin. We analyzed lung gene expression two days after RV infection. For *in vitro* studies, lung CD11c+ cells were isolated from C57BL/6J or Clec9a^gfp-/-^ mice and incubated with hyperoxic BALF and RV. Cells were analyzed by flow cytometry. In neonatal mice, gelsolin blocked hyperoxia-induced Il12p40, TNF-α and IFN-γ mRNA and protein expression in response to RV infection. Similar effects were observed when gelsolin was co-administered with hyperoxic BALF and RV. Gelsolin decreased F-actin levels in hyperoxic BALF *in vitro* and inhibited hyperoxia-induced D103lo DC expansion and inflammation *in vivo*. Gelsolin also attenuated hyperoxia-induced hypoalveolarization. Further, incubation of lung CD11c+ cells from WT and Clec9a^gfp-/-^ mice with hyperoxic BALF and RV, showed Clec9a is required for maximal hyperoxic BALF and RV induced IL-12 expression in CD103+ DCs. Finally, in tracheal aspirates from mechanically ventilated human preterm infants the F-actin to gelsolin ratio positively correlates with FiO2, and gelsolin levels decrease during the first two weeks of mechanical ventilation. Collectively, our findings demonstrate a promising role for gelsolin, administered by inhalation into the airway to treat RV-induced exacerbations of BPD and prevent chronic lung disease.

## Introduction

Prematurity and bronchopulmonary dysplasia (BPD), a chronic lung disease that affects preterm-born infants, are associated with chronic asthma-like symptoms, including recurrent wheezing and airflow obstruction lasting into adulthood (1–4). Despite evidence of reversible airways obstruction (5) and airways hyperresponsiveness to methacholine in children born premature or with BPD (6), the lack of association between chronic respiratory symptoms and atopy (7–9) points to a nonatopic mechanism. The histopathology of the “new”, postsurfactant BPD includes hypoalveolarization, little or no alveolar fibrosis and absence of airway structural changes (10, 11), but these do not fully explain persistent airway inflammation (12) and vulnerability to respiratory viral infection in survivors of prematurity (13, 14). Infection with rhinovirus (RV) is usually mild and self-limited in most infants (15), but often causes severe lower respiratory tract illness, chronic respiratory symptoms, and increased healthcare use in infants with prematurity and BPD (13, 16, 17). The mechanisms responsible for these distinctive responses remain to be defined but may represent a phenomenon of priming the lung immune system by exposures occurring in early life.

In infants with BPD, the duration of supplemental oxygen use is a risk factor for chronic respiratory symptoms (3, 18). Exposure to supraphysiologic oxygen levels, i.e. hyperoxia induces lung injury and inflammation (19). The inflammatory response during hyperoxia could result, at least in part, from immune mediators released from necrotic cells (20–22). Necrotic cell debris exacerbate inflammation (23), stimulate DC maturation and activation, and enhance antigen-stimulated T cell responses (24, 25). Among lung DCs, CD103+ DCs are phagocytic antigen-presenting cells that selectively express Clec9a, a damage-associated molecular pattern (DAMP) receptor for exposed necrotic cell cytoskeletal filamentous actin (F-actin) (26, 27). In the presence of necrotic cells, Clec9a is required for maximal antiviral CD8+ T cell responses and IFN-γ production (28). Co-stimulation of Clec9a and Toll-like receptor 3 (TLR3), a receptor for double-stranded RNA, enhances DC maturation and Th1 cell differentiation (29, 30). The distinctive capacity of lung CD103+ DCs to respond to DAMPs (F-actin) *via* Clec9a and viral infection *via* TLR3 provides a plausible mechanism for priming the inflammatory immune responses to RV infection. Using a mouse model of BPD, we have shown that early-life hyperoxic exposure increases lung IL-12-producing Clec9a+CD103+ dendritic cells (DCs), pro-inflammatory responses and airway hyperreactivity following RV infection (31). We have also demonstrated that neonatal hyperoxia increases the dead cell number and F-actin levels in bronchoalveolar lavage fluid (BALF), and that CD103+ DCs and Clec9a are required for hyperoxia-induced inflammatory responses to RV (32). These results implicate necrotic cell F-actin in hyperoxia-induced inflammatory responses to RV.

Actin release from dead cells is a marker of tissue damage and correlates with the extent of injury (33, 34). Plasma contains two abundant actin-binding proteins, gelsolin and Gc protein (also known as vitamin D-binding protein), that act to depolymerize F-actin (gelsolin) and sequester G-actin (gelsolin and Gc protein) (33). The gelsolin and Gc protein actin-scavenger system can be overwhelmed during conditions in which massive cell injury is present (33, 34). Plasma gelsolin levels are reduced in patients with acute lung injury, burn injury, Alzheimer’s disease, and multiple sclerosis (35–40). Low plasma gelsolin levels are also observed in preterm infants who died or developed BPD, compared to survivors without BPD (41). Gelsolin enhances host defense, reduces neutrophilic inflammation, and improves survival in a mouse model of primary pneumococcal pneumonia (42) and sepsis (43). Additionally, gelsolin mitigates against oxidative damage due to radiation (44), thermal (45) or chemical injury (46). Gelsolin depolymerizes F-actin decreasing its binding to Clec9a in an *in vitro* system (47). Secreted gelsolin inhibits Clec9a-dependent cross presentation of antigen and dampens CD8+ T cell responses in a cancer model in mice (48). However, the role of gelsolin in neonatal hyperoxia-induced, Clec9a+CD103+DC-mediated lung pro-inflammatory responses has not been established.

In this study, we examined the effects of gelsolin treatment on neonatal hyperoxia-induced lung CD103+ DC expansion and inflammatory responses to RV infection, as well as hypoalveolarization. We found that recombinant human plasma gelsolin blocks neonatal hyperoxia-induced expansion of a subpopulation of lung CD103+ DCs, prevents the effects of hyperoxia on pro-inflammatory responses to RV infection and preserves alveolarization. Additionally, we identified a primary role for F-actin, present in hyperoxic BALF supernatant to promote inflammatory responses to RV infection in neonatal mice, and for gelsolin to block these responses.

## Methods

### Human Tracheal Aspirate Collection

We examined tracheal aspirates from infants admitted to the C.S. Mott Children’s Hospital Newborn Intensive Care Unit, as approved by the University of Michigan Institutional Review Board. Entry criteria included gestational age at birth ≤ 32 weeks, mechanical ventilation for respiratory distress, and age ≤ 7 days. Aspirates were collected during routine tracheal suctioning of mechanically ventilated premature infants in the first two weeks of life as described (49). Specimens were centrifuged (1,200 X g for 5 min at 15°C) and supernatants were stored at -80°C.

### Measurement of Tracheal Aspirate F-Actin and Gelsolin Levels

Tracheal aspirates were assayed for extracellular F-actin and gelsolin present in the supernatant using ELISA (F-actin ELISA from MyBioSource, gelsolin ELISA from LSBio).

### Animal model

The animal experiments were performed in strict accordance with the NIH Guide for the Care and Use of Laboratory Animals recommendations. The protocol was approved by the University of Michigan Committee on Use and Care of Animals. Two day-old wild type C57BL/6J, Batf3^-/-^ (B6.129S(C)-Batf3^tm1Kmm^/J) or Clec9a^gfp -/-^ (B6.Cg-Clec9a^tm1.1Crs^/J) mice (Jackson Laboratories, Bar Harbor, ME) were exposed to air or 75% oxygen for up to 14 days using a polypropylene chamber coupled to an oxygen controller and sensor (BioSpherix, Lacona, NY) (50). Pups were produced by breeding homozygous pairs of each genotype. C57BL/6J, as the inbred strain is an approximate control for Batf3^-/-^ and Clec9a^gfp -/-^ mice. Dams were exchanged between air and hyperoxia daily. In selected experiments, neonatal mice were treated with 10ul of PBS (Sigma D6662) or recombinant human plasma gelsolin (Cytoskeleton Inc., HPG6) 0.5mg/kg intranasally daily during exposure to hyperoxia. Intranasal administration of the liquid to anesthetized mice allows for inhalation into the airway. Starting on day of life (DOL) 2, the hyperoxic exposure was continued for 10 days. On DOL 14 the mice were inoculated with 30 µl of RV1B (3 x 10^8^ PFU/ml). Lungs were analyzed immediately after hyperoxia or 2 days after sham or RV infection. Neonatal mice of both sexes were used in all experiments.

### Generation of RV

RV1B (from American Type Culture Collection, Manassas, VA) was grown in HeLa cells, concentrated, partially purified, and titered as described (51). Viral titers were measured by plaque assay (52).

### F-Actin and Gelsolin Levels in Mouse Bronchoalveolar Lavage Fluid

Neonatal mice were exposed to normoxia or hyperoxia for 14 days. On day 14 of exposure, bronchoalveolar lavage (BAL) was performed with 0.3 mL cold PBS. BAL cells were separated from supernatant by centrifugation. F-actin and gelsolin levels in BALF supernatants were measured by ELISA. BALF supernatant (100 μl) was incubated with or without human gelsolin (5 µg) *in vitro* in room temperature for 10 min and F-actin levels were measured by ELISA. In selected experiments, 14-day-old wild type mice were inoculated with hyperoxic BALF 15 µl intranasally, followed by 15 µl of RV1B (3 x 10^8^ PFU/ml). Lungs were analyzed 2 days after RV infection.

### Lung CD11c+ Immune Cells Purification, *In Vitro* Treatment, and Flow Cytometry Analysis

Lung CD11c+ immune cells were purified using CD11c microbeads kit (Miltenyi Biotec, Auburn, CA). Briefly, single cell suspension from five lungs of wild type or Clec9a^gfp -/-^ adult mice were each pooled, incubated with anti-CD11c antibody and purified with the microbeads and columns. The purified lung CD11c+ immune cells were divided into different treatment groups with 1.5X10^6^ cells per condition each time. The purified lung CD11c+ cells were co-cultured with culture medium (10%FBS-DMEM), culture medium containing 20% of hyperoxic BALF, or hyperoxic BALF and RV1B (15 µl of 3 x 10^8^ PFU/ml) overnight in 37°C incubator. After co-culture, the cells were stained with flow antibodies of F4/80, CD11c, CD103, CD11b and IL-12 (BioLengend, San Diego, CA). The fixable live/dead staining is applied to distinguish the live cells (31). The cells were analyzed by flow cytometry machine (BD LSRFortessa) and results were analyzed by FlowJo software.

### Quantitative Real-Time PCR

Mouse whole-lung RNA was prepared using TRIzol (Invitrogen, Carlsbad, CA). Gene mRNA expression (IL-12p40, IFN-γ, TNF-α) was quantified using SYBR green real-time quantitative PCR technology. Primer sequences are listed in **Supplemental Table I**. The level of gene expression was normalized to mRNA of β-actin or glyceraldehyde 3-phosphate dehydrogenase (GAPDH) as indicated, using the 2^-ΔCT^ algorithm. For graphic representation, normalized mRNAs for the target genes were plotted multiplied by 10^-3^.

### Measurement of Cytokines

Whole mouse lung homogenates in PBS were centrifuged and supernatants analyzed for pro-inflammatory cytokines. IL-12p40, IL-12p70, TNF-α and IFN-γ were measured by ELISA (all from R&D Systems, Minneapolis, MN).

### Flow Cytometry

Lungs were perfused with PBS containing EDTA (0.5 mM), minced, and digested with Liberase TM (100 µg/mL; Roche, Indianapolis, IN), together with collagenase XI (250 µg/mL), hyaluronidase 1a (1 mg/mL), and DNase I (200 µg/mL; Sigma, St. Louis, MO) for 1 hour at 37°C (53). Cells were filtered and washed with RBC lysis buffer (BD Biosciences, Franklin Lakes NJ) and kept on ice in media containing 10% serum. Dead cells were stained with Pac-Orange Live/Dead fixable dead staining dye (Invitrogen). Lung cells were then stained with fluorescent-labeled antibodies against various leukocyte surface markers (CD45, CD11b, CD11c, F4/80, CD103, and IL-12). Appropriate isotype-matched controls and Fluorescence Minus One (FMO) controls were used in all experiments. Antibodies were purchased from EBiosciences (San Diego, CA) or Biolegend (San Diego, CA). Cells were fixed and analyzed on a Fortessa (Becton-Dickinson, San Jose, CA) or FACSAria II (BD Biosciences) flow cytometer. Results were analyzed using FlowJo software (Tree Star, Ashland, OR). For analysis of intracellular IL-12 or IFN-γ, fresh aliquots of digested lung tissue were stimulated for 4 h at 37°C with Cell Stimulation Cocktail plus protein transport inhibitors (40.5 µmol/L PMA, 670 µmol/L ionomycin, 5.3 mmol/L brefeldin A, and 1 mmol/L monesin [Invitrogen]), fixed, permeabilized with Cell Permeabilization Buffer (Invitrogen), and incubated with anti-mouse IL-12 clone C17.8 (BioLegend).

### Lung Histology and Morphometry

Lungs were perfused with 5 mM EDTA, inflated to 30 cmH2O pressure with 4% paraformaldehyde (Sigma-Aldrich, St. Louis, MO), and paraffin embedded. Five μm-thick paraffin sections were stained with Hematoxylin and eosin (H&E). To assess alveolarization, alveolar chord length was determined as described (54). In summary, alveolar chord length was calculated as the mean length of line segments on random test lines spanning the airspace between intersections of the line with the alveolar surface was calculated. Four random images from two sections for each animal were photographed at X200.

### Statistical Analysis

Unless otherwise noted, data are represented as mean ± standard error. Statistical significance was determined by unpaired two-tailed *t*-test or one-way analysis of variance, as appropriate. Statistical significance was defined as *P* < 0.05.

## Results

### In a Mouse Model of BPD, Gelsolin Blocks Neonatal Hyperoxia-Induced Inflammatory Responses to RV Infection

We have previously shown that hyperoxic exposure of neonatal mice (a model of BPD) increases the number of activated lung IL-12-producing, Clec9a+CD103+ DCs, induces lung pro-inflammatory responses and airway hyperreactivity following RV infection (31). We have also shown that CD103+ DCs and Clec9a signaling are required for hyperoxia-induced inflammatory responses to RV (32). In addition, we have found that hyperoxia induces airway cell death and increases F-actin levels in BALF supernatant (32). Since gelsolin depolymerizes F-actin decreasing its binding to Clec9a (47), we examined the effects of gelsolin on neonatal hyperoxia-induced inflammatory responses to RV infection.

**Figure 1:**
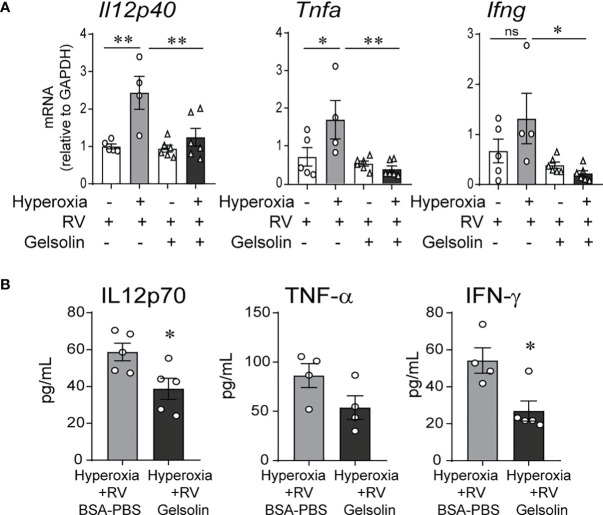
In neonatal mice, gelsolin blocks hyperoxia-induced pro-inflammatory responses to RV infection. Two-day old wild type mice were exposed to hyperoxia or normoxia for 10 days and treated with recombinant human plasma gelsolin (GSN, 0.5 mg/kg) or equal volume BSA-PBS, administered intranasally under anesthesia daily. On day of life 14 the mice were inoculated with RV. Whole lung mRNA expression of *Il12p40, Tnfa and Ifng* **(A)** and protein expression of Il12p70, TNF-α and IFN-γ **(B)** were measured 2 days later. (*N* = 4-6, mean ± SEM, *p < 0.05, **p < 0.01, NS, nonsignificant, ANOVA). One of two independent experiments is shown.

We exposed 2-day-old C57BL/6J mice to normoxia or 75% oxygen for 10 days and administered human gelsolin or control intranasally. Two days after the exposure completed, mice were inoculated with RV intranasally. We have previously published extensive data on sham controls for normoxia- and hyperoxia-exposed neonatal mice (32). Our goal for this study was to compare the effects of gelsolin on normoxia- and hyperoxia-exposed, RV infected mice, therefore we chose not to include a sham control for this study. We found that in RV-infected mice, prior hyperoxic exposure induced type 1 cytokine expression, including Il12, Ifng and Tnfa mRNA. This response was blocked by gelsolin treatment (**Figure 1A**). Protein expression of IL12p70, IFN-γ and TNF-α were also blocked by gelsolin treatment compared with PBS control (**Figure 1B**). These results indicate that gelsolin blocked hyperoxia-induced pro-inflammatory response to RV.

### Gelsolin Inhibits Neonatal Hyperoxia-Induced CD103+ DC Expansion and Inflammation

**Figure 2:**
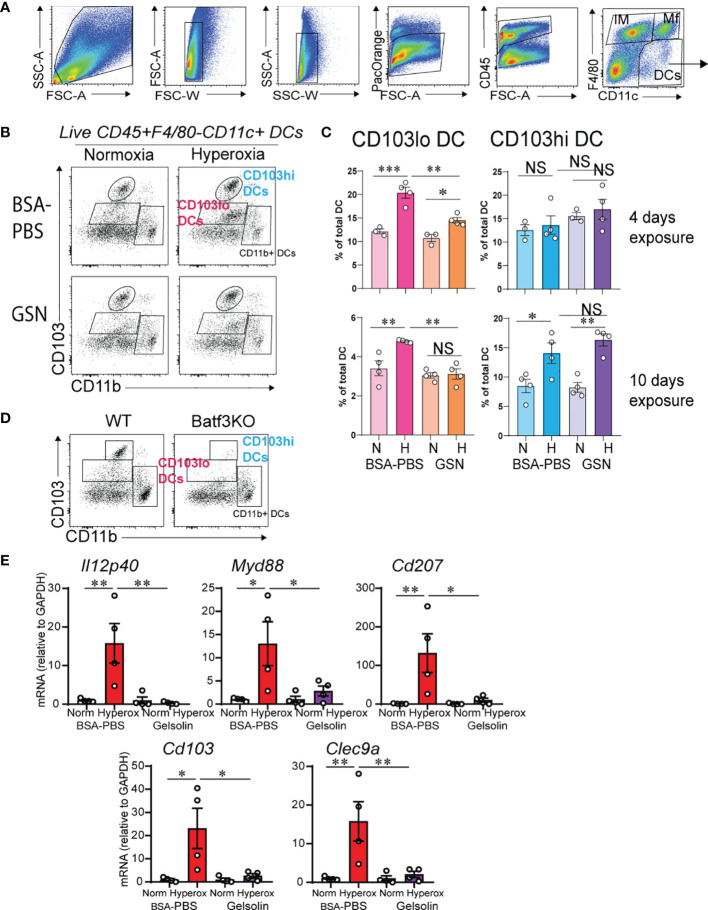
Gelsolin inhibits hyperoxia-induced CD103+ DC expansion and inflammation. Two-day old wild type mice were exposed to hyperoxia or normoxia for 4 or 10 days and treated with recombinant human plasma gelsolin (GSN, 0.5 mg/kg) or equal volume BSA-PBS, administered intranasally under anesthesia daily. Lungs were enzymatically digested, and a single cell suspension was incubated and stained with specific cell-surface antibodies. Lung CD103+ DCs were distinguished from other lung cells based on expression of CD45, F4/80, CD11c, CD103 and CD11b. **(A)** Gating strategy to identify lung DCs. **(B)** Conventional lung DC populations are distinguished based on the expression of CD103 and CD11b. Following hyperoxia, two distinct populations of CD103+ DCs, CD103lo and CD103hi DCs are observed. GSN treatment during hyperoxia decreases CD103lo, but not CD103hi DCs. **(C)** Quantification of the CD103lo and CD103hi DCs. **(D)** Both CD103lo and CD103hi DC populations are absent in the lungs of 4-day old Batf3 null mice compared to age-matched wild type (WT) mice. *p < 0.05, **p < 0.01, ***p < 0.001, NS, nonsignificant (ANOVA). These results are representative of three independent experiments. **(E)** RNA was extracted from the whole lung tissue on day of life 16 after 10 days exposure to normoxia or hyperoxia with and without daily GSN treatment. GSN attenuated hyperoxia-induced mRNA expression of Il12p40, Myd88, Cd207, Cd103 and Clec9a. *p < 0.05, **p < 0.01 (ANOVA). N = 4 per groups. One of three independent experiments is shown.

Based on our previous findings that CD103+ DCs expand and mediate inflammatory gene expression during hyperoxia (31), we examined the effects of gelsolin treatment on lung CD103+ DCs. Recent reports describe two phenotypically and functionally distinct lung migratory CD103+ DC populations following respiratory viral infection, CD103lo and CD103hi DCs (55, 56). Both populations are absent in Batf3-/- mice, that lack CD103+ DCs in lungs and other organs, and unlike CD103hi DCs, CD103lo DCs express lower levels of lineage and maturation markers, including costimulatory molecules, suggesting they are phenotypically immature and functionally limited (55, 56). We looked for evidence of CD103hi and CD103lo DC populations in the lungs of neonatal mice during hyperoxia. Using flow cytometry, we examined lungs of control-treated hyperoxia- and air-exposed neonatal mice after a 4- and a 10-day exposure. These timepoints were designed to assess the acute and chronic responses. After a 4-day exposure, hyperoxia-exposed lungs had a significantly higher number of CD103lo DCs and no change in CD103hi DCs (**Figures 2A–C**). After a 10-day exposure, hyperoxia-exposed lungs had a significant increase in both CD103lo and CD103hi DCs (**Figures 2A–C**). The CD103lo and CD103hi DC populations were not present in lungs of age-matched Batf3-/- mice, confirming that both CD103lo and CD103hi DC populations are Batf3-dependent (**Figure 2D**). Next, we examined the effect of gelsolin on the two lung CD103+ subpopulations during normoxia or hyperoxia. Gelsolin attenuated the effect of hyperoxia on CD103lo DC expansion both after 4- and 10-day exposure, but it did not influence the hyperoxia-induced expansion of CD103hi DCs after 10-day exposure (**Figures 2B, C**). Taken together, these results show that the frequency of CD103hi and CD103lo DCs in neonatal lungs increases during hyperoxia and gelsolin treatment blocks the effect of hyperoxia on CD103lo DCs.

We examined the effects of gelsolin on hyperoxia-induced proinflammatory gene expression. In PBS treated mice, hyperoxia induced the mRNA expression of Il12p40, Myd88, Cd207, Cd103 and Clec9a (**Figure 2E**). Gelsolin treatment attenuated the effect of hyperoxia on the above pro-inflammatory gene mRNA expression (**Figure 2E**). This could reflect either that CD103lo DCs are the primary mediators of hyperoxia-induced proinflammatory responses, or that gelsolin dampens the proinflammatory activation of CD103hi DCs without affecting the increase in frequency during hyperoxia. Alternatively, gelsolin treatment during hyperoxia may modulate the functional properties of other cells that exert anti-inflammatory properties.

### Gelsolin Attenuates Hyperoxic BALF-Induced Inflammatory Response to RV

**Figure 3:**
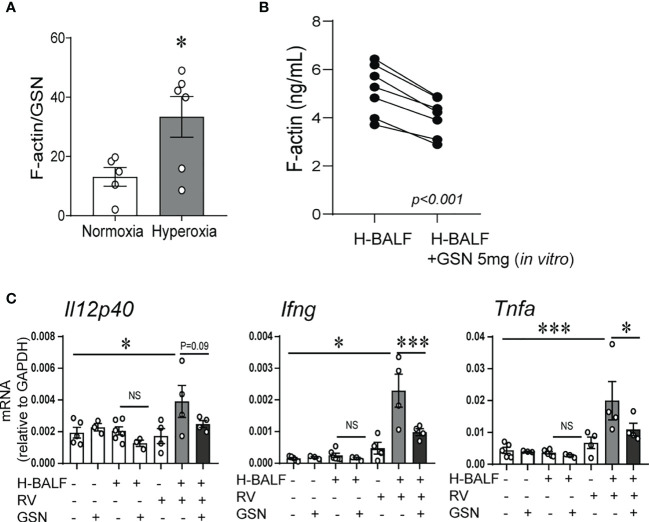
Gelsolin attenuates hyperoxic BALF-induced inflammatory response to RV. Two-day old mice were exposed to hyperoxia or normoxia for 14 days. BALF was collected on day of life 16. F-actin and gelsolin (GSN) protein levels were measured in BALF supernatant using ELISA and the ratio of F-actin to GSN was calculated and compared between samples from hyperoxia- and normoxia-exposed mice, unpaired *t*-test. *p < 0.05 **(A)**. **(B)** 14-day old mice were exposed to hyperoxia for 4 days and BALF was collected (H-BALF). Cell-free H-BALF was incubated with GSN (5mg) *in vitro* for 10min and F-actin levels were analyzed by ELISA (paired t-test *p* < 0.001). **(C)** 14-day old mice were inoculated with H-BALF, RV, GSN, or appropriate controls and whole lung mRNA expression of *Il12p40, Ifng and Tnfa* were analyzed 2 days later. *p < 0.05, ***p < 0.001 (ANOVA). N = 3-6 per group. One of two independent experiments is shown. ns, not significant

We have previously shown that hyperoxic exposure of neonatal mice increases F-actin levels in BALF supernatant (32). To assess the capacity of the actin-scavenger system in the airways, we evaluated the effect of hyperoxia on the balance of F-actin and gelsolin protein levels in the BALF supernatant. To do this we measured F-actin and gelsolin levels in BALF supernatants and calculated the ratio of F-actin to gelsolin levels. The ratio of F-actin to gelsolin levels was significantly higher in hyperoxic BALF (**Figure 3A**). Next, we conducted an *in vitro* experiment to assess if gelsolin can decrease the concentration of F-actin in hyperoxic BALF. Incubation of hyperoxic BALF with gelsolin decreased F-actin concentration (**Figure 3B**). We also designed an *in vivo* experiment and inoculated neonatal mice with hyperoxic BALF and RV, followed by gelsolin, all administered intranasally on DOL14. Whole lung mRNA expression of Il12p40, IFN-γ and TNF-α was assessed 2 days later. The expression of Il12p40, IFN-γ and TNF-α was most significantly upregulated by the combined treatment with hyperoxic BALF and RV and gelsolin attenuated this effect (**Figure 3C**). All other treatments had very small, nonsignificant effects. These results demonstrate that hyperoxia-induced F-actin in BALF plays a key role in mediating proinflammatory responses, especially in the presence of RV.

### Clec9a Is Required for Hyperoxic BALF-Induced Lung CD103+ DC Activation and IL-12 Production

**Figure 4:**
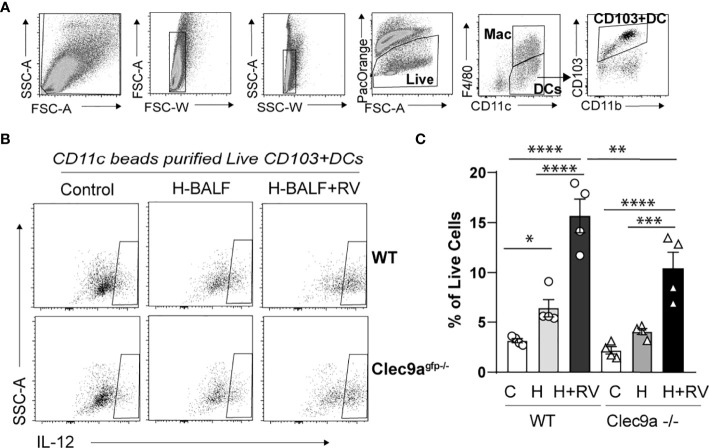
Clec9a is required for maximal hyperoxic BALF-induced pro-inflammatory activation of lung CD103+ DCs. CD11c+ immune cells were purified from wild type (WT) or Clec9a^gfp-/-^ mouse lungs. Equal number of cells were co-cultured with medium and hyperoxic BALF (H-BALF) or H-BALF and RV. Subsequently the cells were stained with antibodies for flow cytometry analysis. **(A)** Gating strategy to identify CD103+ DCs. **(B)** IL-12 expression in CD103+DCs was examined. **(C)** Compared to control **(C)**, the percentage of wild type IL-12+CD103+ DCs was increased after co-culture with H-BALF **(H)** and further increased after co-culture with H-BALF and RV (H+RV). These responses were attenuated in Clec9a null cells. *N* = 4 per group. *p < 0.05, **p < 0.01, ***p < 0.001, ****p < 0.0001 (ANOVA).

To further confirm that the effect of hyperoxic BALF on proinflammatory responses to RV is dependent on Clec9a, the receptor for F-actin, we isolated lung CD11c+ cells from wild type and Clec9a^gfp -/-^ mice. Subsequently, we incubated the CD11c+ cells with hyperoxic BALF and RV. The population of CD11c+ cells in the lung includes both populations of conventional lung DCs, CD103+ DCs and CD11b+ DCs, and resident alveolar macrophages. After 24 hours the cells were subjected to flow cytometry to quantify activation and IL-12 production in lung CD103+ DCs (**Figures 4A–C**). In wild type CD11c+ cells, hyperoxic BALF increased the frequency of IL-12+CD103+ DCs and addition of RV further increased the frequency of these cells. In Clec9a^gfp -/-^ CD11c+ cells, the effect of hyperoxic BALF on IL-12+CD103+ DCs was smaller and nonsignificant and the IL-12 responses of these cells to RV were also attenuated (**Figures 4B, C**). These results indicate that Clec9a expression on CD103+ DCs is required for maximal proinflammatory responses to RV in the presence of hyperoxic BALF, though the remaining response suggests that other signaling pathways contribute to proinflammatory activation of the CD103+ DCs under these conditions.

### Gelsolin Prevents Neonatal Hyperoxia-Induced Hypoalveolarization

**Figure 5:**
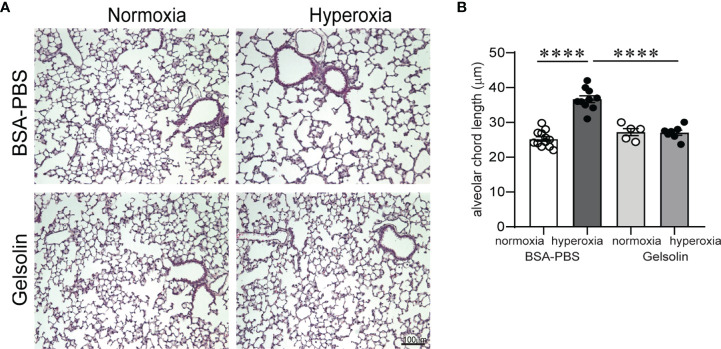
Gelsolin prevents neonatal hyperoxia-induced hypoalveolarization. Two-day old wild type mice were exposed to hyperoxia or normoxia for 10 days and treated with recombinant human plasma gelsolin (0.5 mg/kg) or equal volume BSA-PBS, administered intranasally under anesthesia daily. Lung histology was assessed on day of life 16. **(A)** Representative lung sections were stained with hematoxylin and eosin. Hyperoxia induced enlargement of the alveolar spaces (hypoalveolarization) in BSA-PBS-treated mice. In contrast, gelsolin treatment preserved the alveolar architecture of hyperoxia-exposed mice. **(B)** Alveolar chord length was significantly increased in hyperoxia-exposed, BSA-PBS-treated mice, consistent with hypoalveolarization. In contrast, alveolar chord length of hyperoxia-exposed, gelsolin-treated mice was similar to that of normoxia-exposed mice, indicating protective effect of gelsolin on alveolarization during neonatal hyperoxia. ****p < 0.0001 (ANOVA). N = 5-12 per group.

Inflammation and hypoalveolarization are key histopathologic features that occur in parallel in infants with BPD (10, 11, 57). In neonatal mice, exposure to hyperoxia in early life recapitulates the inflammatory and structural changes associated with human BPD (31, 58). The fact that gelsolin dampened proinflammatory gene expression during hyperoxia prompted us to examine the effects of gelsolin on hyperoxia-induced hypoalveolarization. We administered recombinant human plasma gelsolin (Cytoskeleton,Inc) 0.5 mg/kg intranasally daily during hyperoxic exposure. Hyperoxia was continued for 10 days, starting on DOL 2. Mouse lungs were assessed on DOL 16. In the BSA-PBS (control)-treated mice, as shown previously (58), hyperoxic exposure caused the development of fewer and larger air spaces compared to air-exposed mice (**Figure 5A**). Air exposed gelsolin-treated mice showed a normal alveolarization pattern. Unlike BSA-PBS-treated mice, hyperoxia-exposed gelsolin-treated mice did not show larger air spaces (**Figure 5A**). Differences in alveolarization were quantified using alveolar chord length measurements (**Figure 5B**). These results show that gelsolin treatment during hyperoxia preserves alveolarization.

### In Tracheal Aspirates of Mechanically Ventilated Human Preterm Infants With Respiratory Distress, the F-Actin/Gelsolin Ratio Positively Correlates With FiO2

**Figure 6:**
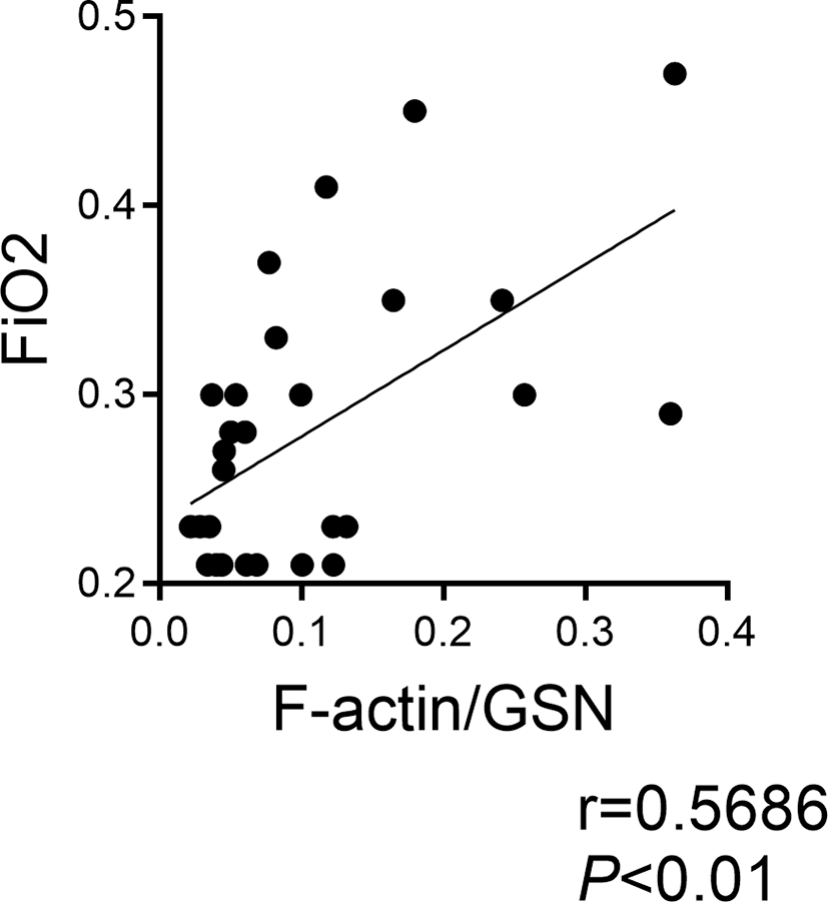
F-actin/Gelsolin ratio in tracheal aspirates of mechanically ventilated human preterm infants with respiratory distress and FiO2 on day of sample collection. Tracheal aspirates were collected in the first week of life. F-actin and gelsolin levels were measured by ELISA in the supernatant. The association between the ratio of F-actin to gelsolin and the FiO2 on the day of sample collection was determined by Pearson’s correlation analysis.

We previously demonstrated that Clec9a+CD103+CD11c+ DCs are present in tracheal aspirates of mechanically ventilated preterm infants in the first week of life, and that tracheal aspirate CLEC9A mRNA levels positively correlated with the proinflammatory cytokine IL12B mRNA expression (32). Since F-actin is a ligand for Clec9a, and F-actin levels are regulated by gelsolin (47), we investigated the relationship between F-actin and gelsolin concentrations in human preterm infant tracheal aspirate supernatants, and the FiO2 on the day of sample collection. We obtained tracheal aspirates from 29 premature infants receiving mechanical ventilation for respiratory distress in the first week of life (**Table 1**). We found that the ratio of F-actin to Gelsolin protein levels positively correlated with FiO2 on the day of sample collection (**Figure 6**). These results demonstrate that F-actin levels relative to Gelsolin levels are increased in preterm infants receiving higher levels of supplemental oxygen.

**Table 1:** Patient characteristics.

n	29
Male gender, *n* (% of total)	17 (59)
Gestational age, mean ± SD, wk	26.4 ± 2.3
Birth weight, mean ± SD, g	968 ± 406
Died, *n* (% of total)	3 (10)
BPD or death, *n* (% of total)	24 (83)
BPD, (% of patients evaluated at 36 wk postmenstrual age), n	21 (81)
FiO2 on day of sample collection, median (IQR)	0.27 ± 0.11
Day of sampling, median (IQR)	3 ± 3

### In Tracheal Aspirates of Human Preterm Infants With Respiratory Distress, Gelsolin Concentrations Decrease During the First Two Weeks of Mechanical Ventilation

**Figure 7:**
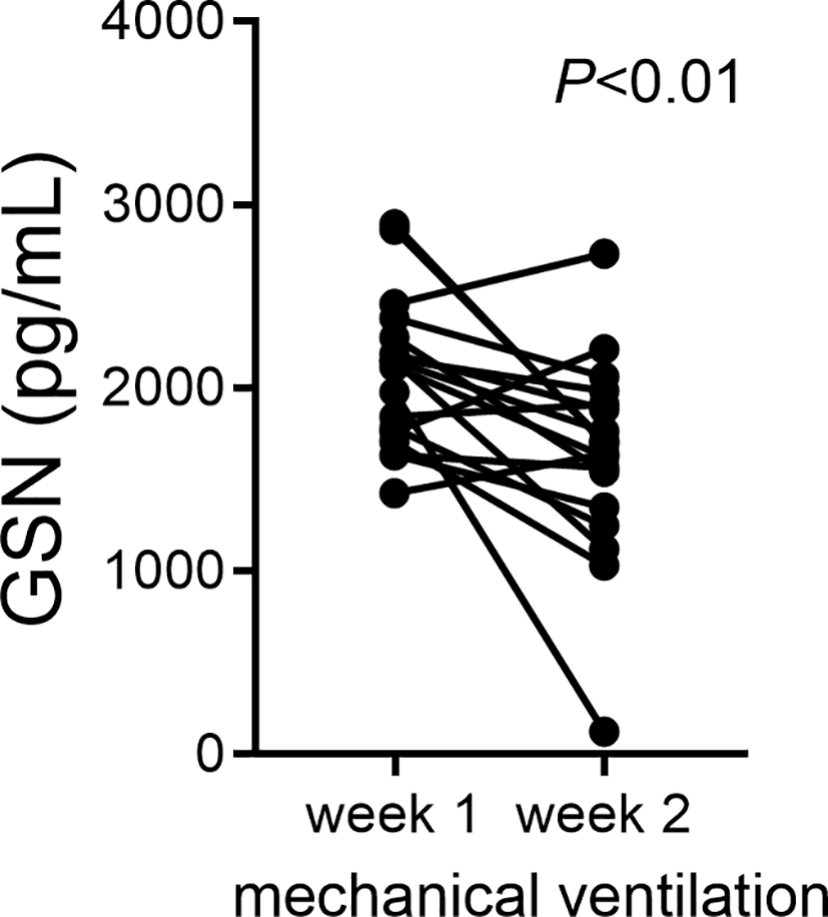
In tracheal aspirates of human preterm infants with respiratory distress, gelsolin concentrations decrease during the first two weeks of mechanical ventilation. For preterm infants who remain endotracheally intubated receiving mechanical ventilation tracheal aspirates were obtained during week 1 and week 2 of mechanical ventilation and gelsolin levels in the supernatant were quantified. Statistical significance was pointed by paired *t*-test.

Cumulative supplemental oxygen exposure during the first two weeks after a preterm birth is a risk factor for BPD development (59). This suggests a critical exposure window that modulates disease development and may involve deficiency of protective mechanisms. We examined changes in gelsolin concentrations in tracheal aspirate supernatants during the first two weeks of life in premature infants. We found that gelsolin levels decrease between week one and week two of mechanical ventilation (**Figure 7**). These findings demonstrate that prolonged mechanical ventilation of preterm infants is associated with relative deficiency in extracellular gelsolin and development of F-actin-dominated disbalance in the actin-scavenger system in the airways.

## Discussion

Prolonged exposure to hyperoxia and BPD are risk factors for asthma-like symptoms in survivors of prematurity (18, 60). Common respiratory viral infections, including infections with RV often cause more severe disease and further increase the risk of recurrent wheezing and airflow obstruction in children born prematurely (4, 16, 17). Currently, there are no methods to prevent severe viral-induced exacerbations or chronic respiratory symptoms in infants with BPD, and treatment is supportive rather than curative. We previously demonstrated that hyperoxic exposure of neonatal mice increases IL-12 production by Clec9a+CD103+DCs and upon RV infection, hyperoxia-exposed but not air-exposed mice show exaggerated pro-inflammatory responses and airway hyperreactivity (31). Our recent work shows that CD103+ DCs and Clec9a are required for hyperoxia-induced pro-inflammatory responses to RV infection and airway hyperreactivity (32). Additionally, we have found that neonatal hyperoxia is associated with a higher number of dead cells in BALF, and elevated F-actin levels in BALF supernatants (32). When cytoskeletal F-actin is released or exposed on necrotic cells, it acts as a DAMP binding Clec9a on CD103+ DCs to trigger enhanced inflammatory cytokine production (61, 62). Simultaneous stimulation of Clec9a and TLR3 further increases DC co-stimulatory molecule expression and, in the presence of antigen, enhances Th1 cell differentiation (29, 30). Gelsolin, an abundant actin-depolymerizing plasma protein (33) decreases binding of F-actin to Clec9a in an *in vitro* system (47). In an *in vivo* cancer model, secreted gelsolin inhibits Clec9a-dependent cross presentation of antigen and dampens CD8+ T cell responses (48). In this study, we examined the role of gelsolin in neonatal hyperoxia-induced Clec9a+CD103+DC-mediated lung pro-inflammatory responses to RV infection and hypoalveolarization. We found that gelsolin blocks neonatal hyperoxia-induced expansion of the CD103lo subpopulation of lung CD103+ DCs, prevents the effects of hyperoxia on pro-inflammatory responses to RV infection and preserves alveolarization. Additionally, we identified a primary role for F-actin, present in hyperoxic BALF supernatant to promote inflammatory responses to RV infection in neonatal mice, and for gelsolin to block these responses.

Lung CD103+ DCs selectively engulf and transport dead, apoptotic cell debris to the draining lymph node (63). Unlike the lung CD11b^hi^ DCs, CD103+ DCs also selectively express TLR3, a receptor for double-stranded RNA which is required for CD8+ T cell antiviral responses (63, 64). Thus, together with signaling *via* Clec9a, the lung CD103+ DCs have a unique functional property to respond to dead, necrotic cells and viral infections simultaneously (29, 30). Two phenotypically and functionally distinct lung migratory CD103+ DC populations, CD103lo and CD103hi DCs have been identified following neonatal respiratory viral infection (55, 56). CD103lo DCs, induced after neonatal RSV infection, have lower expression of lineage-defining markers, costimulatory molecules CD80, CD86, and CD24 expression and display a lower ability to take up and process antigen or stimulate specific CD8+ T cells (56). We found that the frequency of CD103lo and CD103hi DCs was differentially affected during neonatal hyperoxic exposure. Additionally, we found that gelsolin attenuated the hyperoxia-induced increase in CD103lo, but not CD103hi DCs. Our results also demonstrate that gelsolin inhibited the hyperoxia-induced pro-inflammatory responses in whole lung. Our future studies will focus on examining the effect of hyperoxia on the phenotypic and functional properties of CD103lo and CD103hi DCs in the neonatal lung, as well as the effect of subsequent RV infection on these cells.

We are employing RV infection as a non-specific but physiologic and clinically relevant stimulus to probe the hyperoxia-induced activation of CD103+ DCs, the primary DC subset responsible for TLR3-mediated antiviral responses. We have previously demonstrated that neonatal hyperoxic exposure increases whole lung inflammatory responses to the TLR3 agonist poly(I:C) (31), consistent with the notion that the responses are not specific to RV. Additionally, other TLRs and immune cells have been implicated in the inflammatory responses to viral infections. CD11b^hi^ DCs can induce CD8+ T cell proliferation after influenza virus infection in mice (65). Unlike CD103+ DCs, CD11b^hi^ DCs express TLR2 (63). TLR2 is required for early inflammatory responses to RV and RV-induced inflammasome priming (66, 67). However, these studies were in adult mice and not in neonatal mice. Furthermore, age-dependent differences in the immune responses to RV during the first two weeks of life (68), in the context of developing innate immune cell milieu in the lung, could modify the role of CD103+ DCs in the inflammatory responses to RV.

While studying the effects of gelsolin on hyperoxia-induced inflammation and enhanced responses to RV, surprisingly, we found that gelsolin preserves the alveolar architecture during neonatal hyperoxic exposure. Inflammation is a key feature of evolving and established BPD (57, 69). Thus, this finding is a further affirmation about the link between inflammation and impaired alveolar development and broadens the potential about therapeutic use of gelsolin to prevent BPD development.

We have recently found a significant positive correlation between CLEC9A and IL12B expression in tracheal aspirates of mechanically ventilated premature infants with respiratory distress (32). In the present study the ratio of F-actin to gelsolin in tracheal aspirates of mechanically ventilated premature infants in the first week of life positively correlated with FiO2 on the day of sample collection, and gelsolin levels decreased between week 1 and week 2 of mechanical ventilation. There results indicate that with increasing FiO2, the balance between F-actin and gelsolin is skewed towards F-actin. Also, prolonged mechanical ventilation can result in a deficiency of gelsolin in the airway, thus decreasing the capacity to neutralize F-actin. While our results do not confirm a causal inference, they further implicate F-actin-Clec9a-mediated inflammatory responses and a role for gelsolin deficiency in the development of BPD. The human study has several limitations. FiO2 on the day of sample collection may underestimate the amount of oxygen used since birth. Cumulative oxygen exposure may be a better indicator to assess the relationship, however this data was not available to us. When examining changes in tracheal aspirate gelsolin concentrations during the first two weeks of mechanical ventilation, we may have a selection bias, as only samples from infants who remain intubated are available. The natural course and day to day variability in gelsolin concentration in human preterm airways are unknown.

It is possible that gelsolin preserves alveolarization during hyperoxic exposure through a Clec9a- and CD103+ DC-independent mechanism. In addition to depolymerizing actin, gelsolin binds and modulates the effects of bacterial endotoxin (70). Early-life bacterial infection, such as chorioamnionitis and sepsis are major risk factors for BPD development (71–73). Furthermore, postnatal airway gram-negative bacterial dominance is associated with BPD development (74–76). We recently found that neonatal hyperoxia alters the bacterial communities in the lung with predominance of gram-negative bacteria, a change that correlated with inflammation (77). Our future studies will focus on understanding the mechanism by which gelsolin preserves alveolar structure during neonatal hyperoxia.

Gelsolin, administered systemically, enhances host defense, reduces neutrophilic inflammation and improves survival in a mouse model of primary pneumococcal pneumonia (42), and also mitigates against acute hyperoxic lung injury (78). We administered gelsolin intranasally to anesthetized mice to allow for inhalation of the liquid into the airway, the primary site of cell damage and F-actin release. This mode of delivery ensures achieving maximal concentration of gelsolin at the site of cell injury and F-actin release.

We used recombinant human plasma gelsolin to depolymerize and block the effects of mouse F-actin *in vivo* and *in vitro*. Both actin and gelsolin are highly conserved proteins (79, 80), permitting cross species interaction. During *in vivo* exposure to hyperoxia and with use of hyperoxic BALF, gelsolin may exert other functions not related to regulation of actin structure (81). Furthermore, hyperoxic BALF likely contains multitude of proinflammatory medicators, i.e., cytokines, chemokines, or DAMPs. Our results demonstrated that Clec9a is required for maximal pro-inflammatory activation of lung CD103+ DCs in response to hyperoxic BALF alone or in combination with RV. This confirmed a role for F-actin-Clec9a-induced immune responses during hyperoxia and RV infection.

We conclude that recombinant human plasma gelsolin blocks neonatal hyperoxia-induced inflammatory responses to RV infection and preserves alveolarization in mice. These data provide a new mechanism to block the priming effect of neonatal hyperoxia for enhanced inflammatory responses to RV infection and preserve alveolarization. Inhaled recombinant human plasma gelsolin may be an attractive new treatment for RV-induced exacerbations of BPD or preventive therapy for chronic lung disease of prematurity.

## Data Availability Statement

The original contributions presented in the study are included in the article/**Supplementary Material**. Further inquiries can be directed to the corresponding author.

## Ethics Statement

The studies involving human participants were reviewed and approved by The University of Michigan Institutional Review Board. Written informed consent to participate in this study was provided by the participants’ legal guardian/next of kin. The animal study was reviewed and approved by University of Michigan Committee on Use and Care of Animals.

## Author Contributions

AP and TC contributed to the conception and design of the study. TC, AB, Y-JZ, and CF performed experiments, analyzed data. TC and AP wrote first draft and designed figures. AP and TC reviewed, edited and finalized the manuscript. All authors contributed to manuscript revision and approved the submitted version.

## Funding

This work was supported by NIH grant R01 HL140572.

## Conflict of Interest

The authors declare that the research was conducted in the absence of any commercial or financial relationships that could be construed as a potential conflict of interest.

## Publisher’s Note

All claims expressed in this article are solely those of the authors and do not necessarily represent those of their affiliated organizations, or those of the publisher, the editors and the reviewers. Any product that may be evaluated in this article, or claim that may be made by its manufacturer, is not guaranteed or endorsed by the publisher.
