# Supplementary material for: Gelsolin Attenuates Neonatal Hyperoxia-Induced Inflammatory Responses to Rhinovirus Infection and Preserves Alveolarization
Source: Front Immunol. 2022 Jan 31;13:792716. doi: 10.3389/fimmu.2022.792716 (PMC8842948; doi:10.3389/fimmu.2022.792716)
Supplement: Supplementary file 1 [file Table_1.pdf]

Supplemental Table I. Quantitative real-time PCR primer sequences for mouse mRNA (5' to 3'), Forward (F) and Reverse (R)

GAPDH	F: GTC GGT GTG AAC GGA TTT G<br>R: GTC GTT GAT GGC AAC AAT CTC
Il12p40	F: CTC CTG GTT TGC CAT CGT TT<br>R: GGG AGT CCA GTC CAC CTC TA
Ifng	F: ACTGGCAAAAGGATGGTGACA<br>R: TGGACCTGTGGGTTGTTGAC
Tnfa	F: ATG CAC CAC CAT CAA GGA CTC AA<br>R: ACC ACT CTC CCT TTG CAG AAC TC
Myd88	F: GAGCTGCTGGCCTTGTTAGA<br>R: GCGTTTGTCCTAGGGGGTCA
Clec9a	F: GGCCTCTCAGAAGTGCCAAT<br>R: CCTGGAAGAACTTGATGCCCA
Cd103	F: GGCATTCAGTGGTCTGTGCTA<br>R: CAGTGATCTTCGTCCTGTGGTT
Cd207	F: TAAAGCCAGCGCCTTGAACA<br>R: GCCTTGTAGAGAAACTTTTGTTCCG
